# Editorial: Neuromodulation of Exercise: Impact on Different Kinds of Behavior

**DOI:** 10.3389/fnins.2020.00455

**Published:** 2020-05-08

**Authors:** Henning Budde, Bruna Velasques, Pedro Ribeiro, Hideaki Soya

**Affiliations:** ^1^Faculty of Human Sciences, Medical School Hamburg, University of Applied Science and Medical University, Hamburg, Germany; ^2^Federal University of Rio de Janeiro, Rio de Janeiro, Brazil; ^3^Laboratory of Exercise Biochemistry and Neuroendocrinology, Sport Neuroscience Division of Advanced Research Initiative for Human High Performance (ARIHHP), Faculty of Health and Sport Sciences, University of Tsukuba, Tsukuba, Japan

**Keywords:** neuromodulation, physical exercise, physical training, behavior, mental health

The physiology and anatomy of the brain adapts to changing demands by modulating its functional and structural properties (Budde et al., 2016). Convergent evidence from both human (8 studies in this issue) and animal studies (2 studies in this issue) suggests that enhanced physical exercise facilitates this neuromodulation of certain brain structures and as a result behavioral responses.

This special issue wants to enhance our understanding of the neurobiological mechanisms of a variety of physical activities (see Budde et al., 2015b; Wegner et al., 2020 for differentiating issues). The outcome variables referred to cognitive and motor performance measures, white matter volume as well as growth factors, lactate and cortisol.

Kujach et al. found that acute sprint interval exercise (SIE) increases both the cognitive functions and peripheral neurotrophic factors and discussed the possible involvement of lactate in humans, which is a further mechanistic step of previous study that high-intensity interval exercise improves cognitive function (Kujach et al., 2018). Acute SIE shortened response times for both the Stroop task and TMT A and B. In response to acute SIE, blood lactate levels significantly increased and correlated with increased levels of BDNF, IGF-1, and VEGF. Furthermore, cognitive functions and BDNF are found to be correlated. Therefore, the improvement in cognitive performance following SIE may result from the synthesis or release of neuroprotective proteins modulated by high post-exercise blood lactate concentration.

Wegner et al. investigated the “Effects of Different Types of Exercise Training (ET) on the Cortisol Awakening Response (CAR) in Children.” The acute effects of exercise on cortisol have been evaluated in the past (Wegner et al., 2014a,b,c; Budde et al., 2015a). In a longitudinal study for 10 weeks 71 children (9–10 years old) were randomly assigned to a cardiovascular exercise group (*n* = 27), a motor exercise group (*n* = 23), or a control group (*n* = 21). They trained for 45 min., three times a week. Children who enhanced their cardiovascular fitness over the course of the intervention showed an increased CAR after the intervention time, whereas children who underwent a motor exercise intervention and at the same time gained in motor fitness exhibited a decreased CAR after intervention.

Also in the saliva, Caserta et al. measured proNGF and proBDNF levels in 24 subjects before and after two training interventions of 12 weeks. Taken together “Influence of Quadrato Motor Training on Salivary proNGF and proBDNF” suggest that the two neurotrophins undergo a complex modulation, likely related to the different pathways by which they are regulated. Since variations of these neurotrophins have been previously linked to depression, stress and anxiety (Helmich et al., 2010), this study may have practical implications and aid in understanding the possible physiological mechanisms that mediate improved well-being, and the dynamic change of neurotrophins as a result of training.

The study by Matsui et al. entitled “Tyrosine as a Mechanistic-Based Biomarker for Brain Glycogen Decrease and Supercompensation with Endurance Exercise in Rats: A Metabolomics Study of Plasma,” used a rat model of endurance exercise. They detected 186 metabolites in the plasma, and 110 metabolites changed significantly during and following exhaustive exercise. Brain glycogen levels correlated negatively with plasma glycogenic amino acids (serine, proline, threonine, glutamate, methionine, tyrosine, and tryptophan). In particular, plasma tyrosine as a precursor of brain noradrenaline might be a valuable mechanistic-based biomarker to predict brain glycogen dynamics in endurance exercise.

Also in an animal model “Nerve Growth Factor (NGF) is Responsible for Exercise-Induced Recovery of Septohippocampal Cholinergic Structure and Function” Hall et al. showed that exercise-induced enhancement of NGF within the septohippocampal pathway represents a key avenue for aiding failing septo-hippocampal functioning and therefore has significant potential for the recovery of memory and cognition in several neurological disorders.

The study “The Choice of Sports Affects Mental Rotation Performance in Adolescents” by Pietsch et al. investigates mental rotation performance of adolescent female dancers and soccer players in object-based and egocentric mental rotation tasks using human body stimuli. Contrary to the literature, they didn't find significant higher reaction times and error rates for stimuli presented in front view compared to back view in general but only for egocentric transformations. The results of this study show that specific sports affect individual aspects of mental rotation performance.

Van den Berg, Saliasi, Groot et al. showed in their randomized controlled trail “Improving Cognitive Performance of 9–12 Years Old Children: Just Dance?” that daily 10-min exercise breaks in the classroom for 9 weeks did not improve, nor deteriorate cognitive performance in children, comparable with Ludyga et al. (2019).

Van den Berg, Saliasi, Jolles et al. investigated: Exercise of Varying Durations: No Acute Effects on Cognitive Performance in Adolescents. In sum, contrary to literature (Budde et al., 2010; Niemann et al., 2013) acute exercise bouts with a duration of 10, 20, or 30 min did not improve, but neither deteriorate cognitive performance of young adolescents compared to a sedentary control condition.

A group around Terentjeviene et al. performed a study named: Prefrontal Cortex Activity Predicts Mental Fatigue in Young and Elderly Men During a 2 h “Go/NoGo” Task. They did not use exercise as an intervention but measured cognitive stress and their effect on motor functions and concluded: Because of the greater mental load and (possibly) greater activation of prefrontal cortex during the 2 h “Go/NoGo” task, there was greater mental and neuromuscular performance fatigue in young men than in elderly men. However, contrary to this results Wegner et al. (2014a) found an improving effect of acute psychosocial stress on fine motor skills in High School students.

Findings from the ActiveBrains (*n* = 100) and FITKids2 Projects (*n* = 242) named: Physical Fitness, White Matter Volume and Academic Performance in Children by Esteban-Cornejo et al. showed in a cross-sectional design that cardiorespiratory fitness may positively relate to white matter volume in overweight/obese children, and in turn, academic performance.

The results of this special issue suggest that physical exercise triggers neuromodulation and thereby, enhances an individual's capacity to respond to new demands with behavioral alterations (Gronwald and Budde, 2019). Besides the need for more RCT studies we believe that it will become more and more necessary to implement an extra sham condition in future studies to be able to prove that one exercise intervention is more effective than another (Budde et al., 2018).

## Author Contributions

HB and HS conceptualized and drafted the initial manuscript. HB, BV, PR, and HS reviewed and revised the manuscript. All authors have read and approved the final version of the manuscript.

## Conflict of Interest

The authors declare that the research was conducted in the absence of any commercial or financial relationships that could be construed as a potential conflict of interest. The handling Editor declared a shared affiliation with one of the author HS.
